# Interaction of TPPP3 with VDAC1 Promotes Endothelial Injury through Activation of Reactive Oxygen Species

**DOI:** 10.1155/2020/5950195

**Published:** 2020-10-02

**Authors:** Naijia Liu, Yintao Li, Wu Nan, Wenbai Zhou, Jinya Huang, Rumei Li, Linuo Zhou, Renming Hu

**Affiliations:** ^1^Department of Endocrinology and Metabolism, Huashan Hospital, Fudan University, Shanghai, China; ^2^Department of Oncology, Shandong Cancer Hospital and Institute, Shandong First Medical University and Shandong Academy of Medical Sciences, Jinan, China; ^3^Department of Geriatrics, Zhongshan Hospital, Shanghai Medical College, Fudan University, Shanghai, China; ^4^Department of Medicine, Emanuel Medical Center, Turlock, California, USA; ^5^Institute of Endocrinology and Diabetology, Fudan University, Shanghai, China

## Abstract

Endothelial injury plays a critical role in the pathogenesis of cardiovascular disorders and metabolic-associated vascular complications which are the leading cause of death worldwide. However, the mechanism underlying endothelial dysfunction is not completely understood. The study is aimed at investigating the role of tubulin polymerization-promoting protein family member 3 (TPPP3) in palmitic acid- (PA-) induced endothelial injury. The effect of TPPP3 on human umbilical vein endothelial cells (HUVECs) was determined by evaluating apoptosis, tube formation, and reactive oxygen species (ROS) production. TPPP3 silencing inhibited PA overload-induced apoptosis and production of ROS, along with the alteration of apoptosis-related key proteins such as BCL-2 and Bax. Mechanically, voltage-dependent anion channel 1 (VDAC1) was identified as a novel functional binding partner of TPPP3, and TPPP3 promoted VDAC1 protein stability and its activity. Further studies indicated that TPPP3 could promote apoptosis, ROS production, tube formation, and proapoptotic protein expression and reduce antiapoptotic protein expression through increasing VDAC1 expression under mildly elevated levels of PA. Collectively, these results demonstrated that TPPP3 could promote PA-induced oxidative damage in HUVECs via a VDAC1-dependent pathway, suggesting that TPPP3 might be considered as a potential therapeutic target in vascular disease.

## 1. Introduction

Vascular disease is the top cause of mortality and disability worldwide. Impairment of endothelial function may have dramatically contributed to the initiation and development of vascular diseases [1, 2]. Vascular endothelial cells constitute the inner cellular lining of the blood vessel and are closely regulated by changes in the metabolism. The integrity of endothelial cells helps maintain the overall cardiovascular homeostasis. Endothelial dysfunction not only involves the pathogenesis of various cardiovascular events and vascular complications of diabetes but also is the starting point of these diseases [3–5]. Therefore, the protection of endothelial function has a potential benefit for cardiovascular disorder and diabetic vascular complications. Increasing evidence shows that high levels of reactive oxygen species- (ROS-) induced oxidative stress is a critical pathogenic factor in endothelial cell injury [6, 7]. It is well documented that patients with hyperlipidemia, hypertension, and metabolic syndrome have elevated levels of free fatty acids (FFAs) [8, 9]. In obesity patients, metabolic stress resulting from increased FFAs gives rise to endothelial dysfunction and apoptosis [10, 11], which promotes atherosclerosis and other cardiovascular diseases. However, the exact mechanism of how lipid disorders cause endothelial injuries remains to be defined.

Tubulin polymerization-promoting protein family member 3 (TPPP3), a member of tubulin polymerization-promoting proteins, was identified on chromosome 16q22.1. Our previous studies have shown that TPPP3 was upregulated in non-small-cell lung cancer and colorectal cancer [12, 13]. The following studies suggested that TPPP3 was involved in axon regeneration of zebrafish [14]. Recently, TPPP3 inhibition had been shown to reduce stromal-to-decidual transition in human endometrial stromal cells (hESCs) by suppressing the *β*-catenin/NF-*κ*B/COX-2 signaling pathway [15]. However, the underlying role and mechanism of TPPP3 in endothelial injury has not yet been reported.

Palmitic acid (PA), the main saturated free fatty acid in the human body [16], can induce endothelial “lipotoxicity,” which is considered as the primary cause of endothelial dysfunction and a sign of cardiovascular disease. Previous studies reported that the exposure of endothelial cells to PA leads to cell necrosis [17] and imflammation [9, 18]. It is generally accepted that PA-induced cell injury occurs due to increased ROS or oxidative stress [19, 20]. Thus, endothelial cell exposure to PA is a suitable model for the exploration of the mechanisms of FFA-induced endothelial injury. In our present study, the expression of TPPP3 was determined in human umbilical vein endothelial cells (HUVECs) treated by PA. Then gain- and loss-of-function experiments were performed to assess the biological functions of TPPP3 with or without PA treatment. Following coimmunoprecipitation (Co-IP) combined with aliquid chromatography-masss pectrometry (LC-MS)-based targeted proteomic assay was performed to identify potential TPPP3 interacting partners. Specifically, VDAC1 was found to interact with TPPP3 and was involved in TPPP3-mediated production of ROS and HUVEC injuries.

## 2. Materials and Methods

### 2.1. Cell Culture and Treatment

Human umbilical vein endothelial cells (HUVECs) were obtained from the cell bank of the Chinese Academy of Sciences (Shanghai, China) and cultured in an EGM-2 BulletKit (Lonza, Switzerland) supplemented with 100 U/mL penicillin and 100 *μ*g/mL streptomycin in a humidified atmosphere containing 5% CO_2_ at 37°C. The culture medium was replaced every other day. For palmitic acid (PA, Sigma-Aldrich, MO, USA) exposure experiments, when cells reached 70~80% confluence, they were exposed to serum-free medium containing 0.5% bovine serum albumin (BSA) for 24 h before exposure to various concentrations of PA for the indicated time.

### 2.2. Plasmid Construction, Viral Transfection, and siRNA Transfections

For knocking down of TPPP3, specific short hairpin RNA- (shRNA-) targeting TPPP3 was synthesized and cloned into a pLKO.1-TRC cloning vector (Addgene, MA, USA). The shTPPP3 sequences were 5′-CCGGCTGCTCGGGTCATCAACTATGCTCGAGCATAGTTGATGACCCGAGCAGTTTTTG-3′ (shRNA#1) and 5′-CCGGCATCGTCTTCTCCAAAGTCAACTCGAGTTGACTTTGGAGAAGACGATGTTTTTTG-3′ (shRNA#2). TPPP3 ectopic expression and negative control lentiviruses were purchased from GeneChem (Shanghai, China). Viral transfection was performed as we previously described [12]. HUVECs were transduced with lentivirus-mediated control vector (SCR) or shTPPP3 according to the manufacturer's instructions. The efficiency of knockdown was verified by Western blotting.

Small interfering RNA (siRNA) oligonucleotides specific for VDAC1 (siVDAC1) and negative controls (NC) were synthesized by GeneChem. VDAC1 overexpression and control vector (Vector) were purchased from Santa Cruz Biotechnology (Santa Cruz, CA, USA). Cells were cultured in 6-well plates and transfected using a Lipofectamine RNAiMAX® Transfection Reagent (Invitrogen, CA, USA) according to the manufacturer's instructions.

### 2.3. Flow Cytometry

Following treatment, cells were harvested and washed with cold phosphate-buffered saline (PBS). Then, 5 *μ*L Annexin V-FITC (BD Biosciences, CA, USA) was added and incubated in the dark at room temperature for 15 minutes. After exposure to 10 *μ*L of propidium iodide, the percentages of apoptotic cells were determined using FACSCanto II flow cytometry (BD Biosciences, CA, USA).

### 2.4. DCFDA Assay for Intracellular ROS

The production of ROS was measured by using a 2′7′-dichlorodihydrofluorescein diacetate (DCFH-DA) reagent (Sigma-Aldrich). After treatment as described, cells (1 × 10^5^) were seeded onto coverslips in 6-well plates, and the cells were treated with 5 *μ*M of DCFH-DA for 30 min, and the images of the cells were captured under Olympus FluoView FV1000 (Olympus, Melville, NY, USA).

### 2.5. Tube Formation Assay

Matrigel (BD Biosciences) was diluted with EBM-2 medium containing 0.5% serum and coated in 24-well plates at 37°C for 2 h. HUVECs were suspended at a density of 2 × 10^4^ cells/mL and seeded alone or cocultured with PA on Matrigel. After 6 h of incubation, 6 random fields per sample were photographed at ×100 magnification with a computer-assisted inverted microscope (Nikon).

### 2.6. Immunofluorescence

Cells were seeded in a 24-well plate to adhere for 24 h. After fixing with 4% paraformaldehyde for 15 minutes, the cellular membrane was permeabilized with 0.1% Triton X-100 for 15 min and blocked with 5% BSA for nonspecific binding. Then, cells were incubated with primary antibodies TPPP3 and VDAC1 in a humidified incubator for 24 h at 4°C. After treatments, cells were washed and treated with FITC-conjugated secondary antibody and Alexa Fluor® 555-labeled secondary antibody for 1 hour. Finally, the nuclei were counterstained with DAPI, and fluorescence images were captured using a confocal laser scanning microscope (Nikon, Tokyo, Japan).

### 2.7. Coimmunoprecipitation (Co-IP) Analysis

The cells were lysed with IP buffer (Sigma-Aldrich). After the protein concentration was determined to be 1 mg/mL, the cell lysates were incubated with TPPP3 or VDAC1 antibody, as well as the protein A/protein G-coated agarose beads (Sigma-Aldrich) overnight at 4°C. For control, the same amount of precleared mitotic extract was incubated with mouse nonspecific IgG covalently coupled with Protein G Sepharose. Then, cell lysates were washed with PBS and centrifuged at 1000 × g. The proteins were then separated from the beads using immunoblotting loading buffer for 10 minutes at 95°C. The supernatants were harvested for subsequent immunoblotting analysis with the indicated antibodies.

### 2.8. Cycloheximide Chase (CHX-Chase) Assay

HUVECs were transduced with vector or TPPP3 ectopic expression lentivirus for 48 hours or treated with PA or PBS, then exposed to 10 *μ*g/mL cycloheximide (CHX) for 0, 30, 60, or 120 min. VDAC1 protein residue was determined by Western blot.

### 2.9. Western Blot Analysis

After treatments, cells were lysed in a RIPA lysis buffer (Cell Signaling Technology, MA, USA) containing a tablet of protease inhibitor (Roche, Switzerland). All samples were separated to 10% SDS-PAGE and transferred to PVDF membranes (Millipore, MA, USA). After blocking in 5% skim milk at room temperature, the membranes were incubated with the following primary antibodies overnight at 4°C: TPPP3 (Abcam, MA, USA) and VDAC1, Caspase-3, Caspase-9, Cytochrome C, BCL-2, and Bax all purchased from Cell Signaling Technology, followed by the appropriate peroxidase-conjugated secondary antibody for 2 h at room temperature. The protein bands were visualized using an ECL Substrate Kit (Thermo Fisher Scientific Inc., MA, USA) on an ImageQuant LAS 4000 (GE Healthcare, NJ, USA). GAPDH served as the control of total protein expression.

### 2.10. Statistical Analysis

All data were presented as mean ± standard deviation (SD) of at least three independent experiments. Statistical analysis between groups or among groups was analyzed using Student's *t*-test or one-way ANOVA followed by the post hoc Bonferroni test. *p* value < 0.05 was considered statistically significant.

## 3. Results

### 3.1. PA-Induced TPPP3 Expression

**Figure 1:**
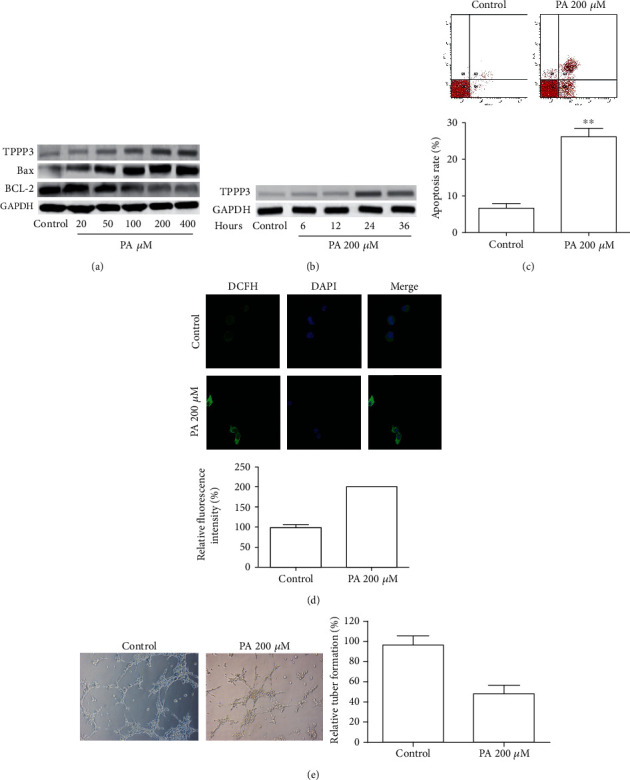
PA increased TPPP3 expression in HUVECs. (a) Representative Western blot analyses of TPPP3, Bax, and BCL-2 expression in HUVECs after 24 h of exposure to palmitic acid. (b) Representative Western blot analyses of TPPP3 expression in HUVECs treated with 200 *μ*M palmitic acid at the indicated time point. (c–e) HUVECs were treated with 200 *μ*M PA for 24 hours; cell apoptosis (c), production of ROS (d), and the relative number of tube branches (e) were measured by flow cytometry, DCFDA assay, and tube formation assay, respectively. Magnification, ×100. Student's *t*-test was performed to assess the difference. ^∗∗^*p* < 0.01.

Previous studies have shown that PA overload-induced oxidative stress is a crucial event in the initial development of endothelial dysfunction [21]. To assess the effect of lipid disorders on the expression of TPPP3 in HUVECs, HUVECs were treated with different concentrations of PA. As shown in Figure 1(a), when the protein levels of TPPP3 increased, apoptosis suppressor protein BCL-2 decreased and proapoptotic protein Bax increased with the stimulation of PA in a dose-dependent manner. Furthermore, the expression of TPPP3 was increased after exposure to 200 *μ*M PA and reached the peak at 24 h (Figure 1(b)). Then, the effects of PA on HUVECs were determined; the apoptosis (Figure 1(c)) and release of ROS (Figure 1(d)) were significantly increased after PA treatment. Besides, tuber formation assay showed that the angiogenic ability of HUVECs decreased after PA treatment (Figure 1(e)). No cytotoxicity was observed in HUVECs at doses below 50 *μ*M of PA (data not shown). These results demonstrated that PA was able to induce lipotoxicity, accompanied by the increase of TPPP3.

### 3.2. TPPP3 Is a Mediator of PA-Induced ROS Generation

**Figure 2:**
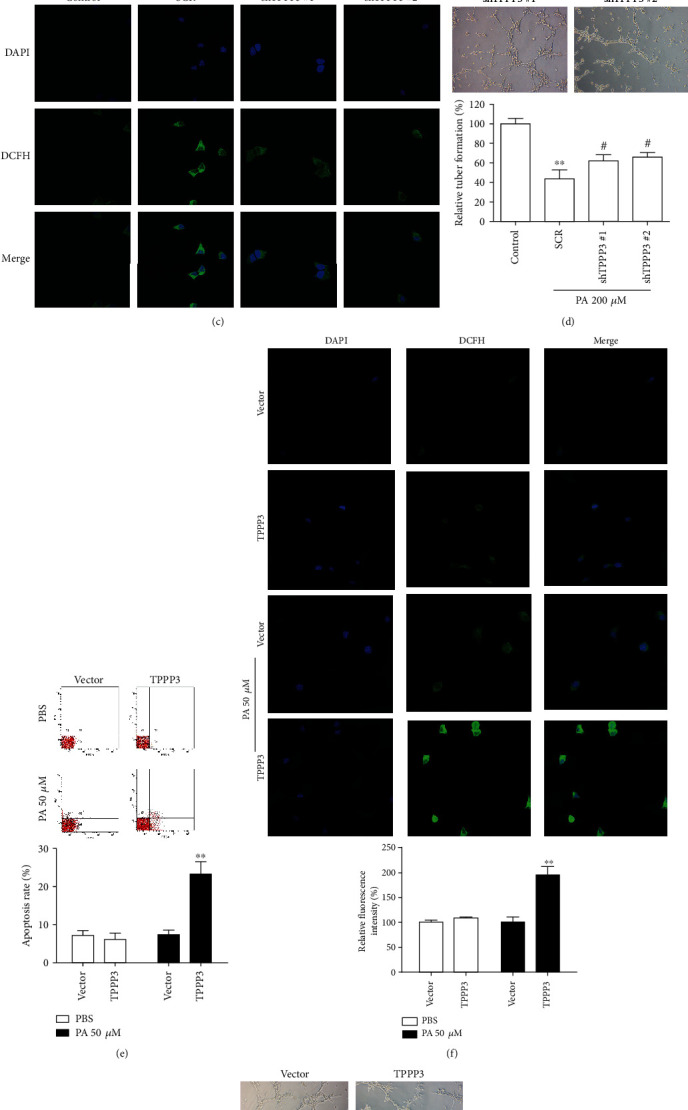
Influence of TPPP3 in PA-induced endothelial injuries in HUVECs. HUVECs were transduced with SCR (scrambled shRNA) or TPPP3 shRNAs and then treated with 200 *μ*M PA for 24 h before the following analyses. (a) TPPP3, BCL-2, Caspase-3, Caspase-9, Cytochrome C, and Bax protein level in HUVECs after TPPP3 silencing measured by Western blot. (b) Flow cytometry analysis, (c) representative images of immunostaining, and (d) Matrigel tube formation assay of HUVECs as indicated treatment. Flow cytometry analysis of cell apoptosis (e) and representative images of ROS staining (f) showed cell apoptosis and production of ROS in TPPP3 overexpressed HUVECs treated with 50 *μ*M PA. (g) Tube formation assay showed overexpression of TPPP3 aggravated PA-induced impairment of endothelial tube formation. Student's *t*-test or one-way ANOVA was performed to assess the difference. ^∗∗^*p* < 0.01 vs. control, ^#^*p* < 0.01 vs. SCR.

Since TPPP3 was induced by PA, the roles of TPPP3 in PA-regulated endothelial injuries were assessed. To investigate the effect of TPPP3 on the biological function of HUVECs, cells were selected to be transduced with TPPP3 shRNAs for further study. The protein levels of TPPP3 were efficiently decreased after the lentivirus infection (Figure 2(a)). TPPP3 silencing decreased PA-induced cell apoptosis and release of ROS, as indicated in Figures 2(b) and 2(c), respectively. Similarly, the knockdown of TPPP3 resulted in an increase of capillary-like tubes inhibited by PA (Figure 2(d)). To further investigate the roles of TPPP3 in endothelial injuries, the biological functions of TPPP3 were determined using gain-of-function in HUVECs. Interestingly, an increase of cell apoptosis (Figure 2(e)) and release of ROS (Figure 2(f)) were observed when TPPP3-overexpressed HUVECs were treated with low PA (50 *μ*M). 50 *μ*M PA treatment TPPP3-overexpressed HUVECs also inhibited tube formation (Figure 2(g)). In contrast, we did not observe significant change after TPPP3 overexpression, including cell apoptosis, release of ROS, and capillary-like tubes (Figures 2(e)–2(g)) under normal state. All these findings supported that TPPP3 was involved in PA-induced apoptosis and the release of ROS in HUVECs under a pathological state.

### 3.3. TPPP3 Induces the Accumulation of ROS by Interfering with VDAC1

**Figure 3:**
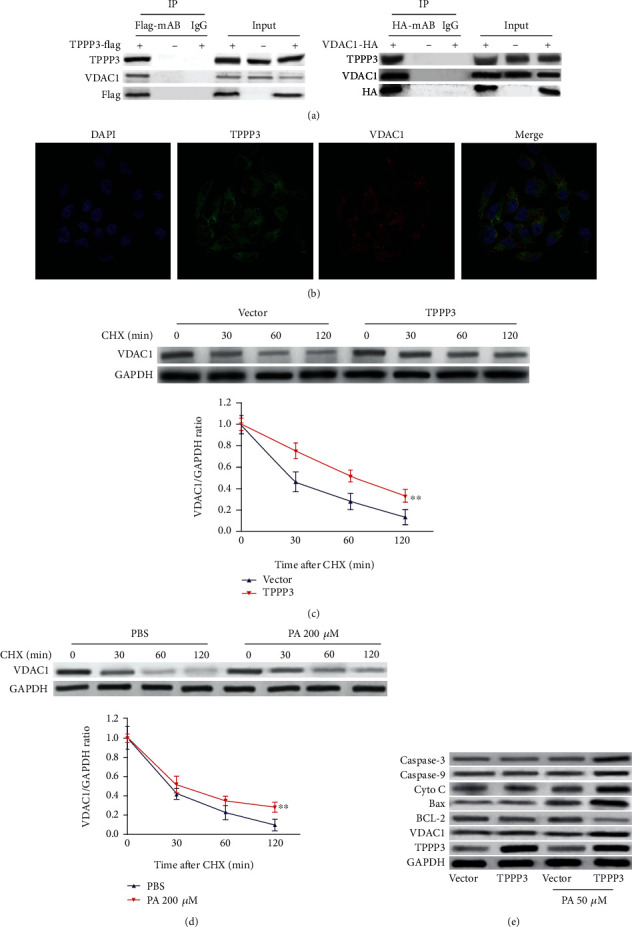
TPPP3 binds and stabilizes VDAC1. (a) Immunoprecipitation (IP) of cell lysates from the HUVECs transfected with TPPP3-FLAG (left) or VDAC1-HA (right) with antibodies to FLAG or HA, and Western blot analysis with the indicated antibodies to TPPP3 or VDAC1. Irrelevant rabbit IgG was used as control. (b) Representative confocal images of TPPP3 (green) and VDAC1 (red) are shown; colocalization of TPPP3 and VDAC1 was observed (yellow) in HUVECs. (c) HUVECs were transduced with vector or TPPP3 and then treated with CHX (10 *μ*g/mL) for the indicated time. The expressions of VDAC1 and GAPDH were determined using Western blotting. (d) HUVECs were treated with 200 *μ*M PA and then treated with CHX (10 *μ*g/mL) for the indicated time. The expressions of VDAC1 and GAPDH were determined using Western blotting. (e) Western blot analysis of TPPP3, VDAC1, BCL-2, Bax, Cyto C, Caspase-3, and Caspase-9 proteins following overexpression of TPPP3 in HUVECs with or without 50 *μ*M PA treatment. One-way ANOVA was performed to assess the difference. ^∗∗^*p* < 0.01.

To analyze the mechanism through which TPPP3 contributes to endothelial injuries, Co-IP combined with LC-MS/MS were performed in HUVECs transduced with vector or TPPP3. Coomassie Brilliant Blue-stained SDS-PAGE gels showed more protein bands in the IP samples from TPPP3 overexpression than in the vector (data not shown). Then, pelleted Co-IPs from HUVECs were subjected to liquid chromatography/mass spectrometry (LC/MS) analysis. A total of 700 proteins were identified as TPPP3 potentially interacting partners (Table S1). MS analysis showed that voltage-dependent anion channel 1 (VDAC1) is ranked as one of the most abundant proteins that bind to TPPP3 in HUVECs. Previous studies have shown that VDAC1 is involved in ROS generation, then VDAC1 is selected for further investigation [22–24]. To determine whether TPPP3 interacts with VDAC1 in HUVECs, Co-IP was performed in the protein lysates of HUVECs. Co-IP/Western blot assay showed that TPPP3 was coprecipitated with endogenous VDAC1 (Figure 3(a), left). In a reciprocal Co-IP experiment, similar results were observed that VDAC1 coprecipitated with TPPP3 (Figure 3(a), right). The colocalization of TPPP3 and VDAC1 was observed by immunofluorescence assay in HUVECs (Figure 3(b)). Since TPPP3 could increase and relocalize VDAC1, we then measured whether TPPP3 affected VDAC1 stability. Interestingly, overexpression of TPPP3 prolonged the half-life of VDAC1 when measured using a CHX-chase assay (Figure 3(c)). Similarly, PA treatment could also prolong the half-life of VDAC1 using a CHX-chase assay (Figure 3(d)). The results of Western blot assay showed that overexpression of TPPP3 upregulated the expression of VDAC1 and proapoptotic proteins (Bax, Cyto C, Caspase-9, and Caspase-3) under low PA treatment (Figure 3(e)). However, without mild PA stimulation, TPPP3 could not exert damage effect. Taken together, these results suggested that TPPP3 could induce endothelial injuries through interfering with VDAC1, whereas this effect required mild PA stimulation (50 *μ*M).

### 3.4. VDAC1 Is Involved in TPPP3-Induced HUVEC Damage

**Figure 4:**
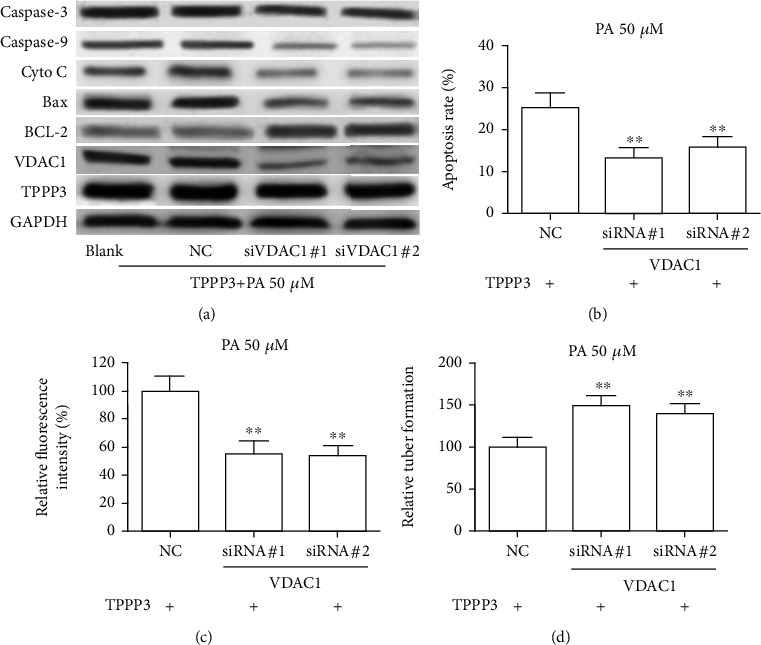
VDAC1 plays a dominant role in TPPP3-induced HUVEC injuries. HUVECs overexpressing TPPP3 were treated with 50 *μ*M PA for 24 hours and then transfected with VDAC1 siRNAs. (a) Protein expression levels of above genes were determined through Western blotting analysis. Cell apoptosis (b), production of ROS (c), and tube formation assay (d) of HUVECs as indicated treatment. One-way ANOVA was performed to assess the difference. ^∗∗^*p* < 0.01.

Since TPPP3 could enhance the stabilization of VDAC1, the roles of VDAC1 in TPPP3-medicated endothelial dysfunction were explored in TPPP3 overexpressed HUVEC. We designed two siRNAs to knock down VDAC1; as expected, siRNA directed against VDAC1 could effectively decrease the expression of VDAC1 (Figure 4(a)). Then, we observed that cell apoptosis (Figure 4(b)), release of ROS (Figure 4(c)), and impairment of capillary-like tubes (Figure 4(d)) induced by TPPP3 under 50 *цM* PA treatment were markedly reversed by VDAC1 knockdown. Consistent with the alteration of biological functions, knockdown of VDAC1 with siRNA inhibited TPPP3-induced Cyto C, Caspase-3 and Caspase-9 upregulation, and BCL-2 downregulation (Figure 4(a)), suggesting the involvement of VDAC1 in TPPP3-induced HUVEC dysregulation.

### 3.5. ROS Scavengers Reverse the Effects of TPPP3 on HUVECs

**Figure 5:**
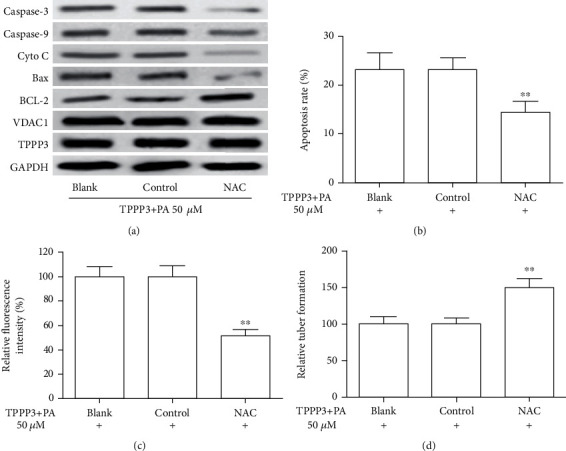
Role of ROS in TPPP3-induced cell dysfunction in HUVECs. HUVECs overexpressing TPPP3 were treated with 5 *μ*M NAC or control or without any treatment for 2 h and then incubated with 50 *μ*M PA for 24 hours. (a) The cell lysates were analyzed by Western blot using antibodies against TPPP3, VDAC1, BCL-2, Bax, Cyto C, Caspase-3, and Caspase-9. Apoptosis analysis (b), production of ROS (c), and capillary-like tubes (d) were detected by flow cytometry, DCFDA assay, and tube formation assay, respectively. One-way ANOVA was performed to assess the difference. ^∗∗^*p* < 0.01.

Then, we studied if ROS played a role in TPPP3-induced HUVEC injuries and the apoptosis signal pathway. Functional studies showed that 5 mM NAC (a ROS scavenger) treatment significantly reversed the TPPP3-induced increase of cell apoptosis (Figure 5(b)) and release of ROS (Figure 5(c)). The capillary-like tubes also rised after NAC treatment (Figure 5(d)). Then, the results of the Western blot analysis demonstrated that expression of BCL-2 was increased, whereas the expression of Bax, Cyto C, Caspase-3, and Caspase-9 was reduced in HUVECs after NAC treatment. However, they had little effect on the expression of TPPP3 and VDAC1 (Figure 5(a)). These results indicated that TPPP3 interacted with VDAC1 to produce ROS and subsequently induce HUVEC apoptosis and dysregulation.

## 4. Discussion

An increasing number of experiments have confirmed that lipid accumulation results in cell apoptosis, oxidative stress, and insulin resistance [25, 26]. Several molecular processes associated with PA in HUVEC pathological processes have been reported, including endoplasmic reticulum (ER) stress, insulin resistance, release of ROS, and inflammation response [27–29]. In the current study, we confirmed the effects of PA on HUVEC damage and examined the expression of TPPP3 in HUVEC exposure to PA. Our data demonstrated for the first time that TPPP3 expression was significantly increased in PA-treated HUVECs and knockdown of TPPP3 protected HUVECs from apoptosis and release of ROS in response to PA. To further investigate the mechanism of TPPP3 involved in apoptosis and damages of HUVECs, the combination of Co-IP and LC-MS analysis was performed to identify the novel TPPP3-associating proteins. VDAC1 was identified as a novel TPPP3-interacting protein. Our work demonstrated that TPPP3 might participate in HUVEC damage under mild PA treatment through regulating the stability of VDAC1 .

Members of the tubulin polymerization-promoting protein (TPPP) family are potent regulators of cell proliferation in most developmental and physiological processes [30]. TPPP3, also known as P20, binds to tubulin, stabilizes and polymerizes microtubules, and displays microtubule-associated protein- (MAP-) like features [30, 31]. Our previous studies demonstrated that TPPP3 could promote the development of colorectal cancer as well as non-small-cell lung cancer [12, 13, 32]. Furthermore, TPPP3 silencing could inhibit the growth of HeLa and Lewis lung carcinoma cells [33, 34]. TPPP3 was also reported to be implicated in glioblastoma multiforme (GBM) progression and prognosis [35]. To our knowledge, this is the first study to explore the role and mechanism of TPPP3 in endothelial injury. Interestingly, a recent study identified that TPPP3 is associated with blood pressure [36] which is a common cardiovascular disease, providing suppport for our findings. Oxidative stress to endothelial cells could lead to disturbance of NO metabolism and endothelial dysfunction, underlying the development and progress of hypertension [37]. In our study, knockdown of TPPP3 in PA-treated HUVECs could significantly prevent HUVECs from apoptosis and release of ROS, providing a new potential target for treatment of cardiovascular disease.

To explore the molecular mechanism of TPPP3, we discovered the interacting partners of TPPP3 via LC-MS/MS-based targeted high-throughput proteomics. Analysis revealed that TPPP3 interacted with VDAC1. Then, Co-IP confirmed the interaction of TPPP3 and VDAC1. VDAC1, as a member of VDACs, was located at the outer membrane of mitochondria and served as a mitochondrial gatekeeper for the entry and exit of mitochondrial metabolites [38, 39]. Previous studies have shown that VDAC1 regulated a variety of cellular processes, such as apoptosis, metabolism, and ion homeostasis [40–42]. Given its location, VDAC1 was able to interact with various proteins that mediate and regulate mitochondrial functions. VDAC1 interacting with hexokinase(HK) forms a polymeric channel that promotes energy accumulation in mitochondria, hence promoting cell growth and survival [43]. VDAC1 has been shown to be a target for both the antiapoptotic (BCL-2 and Bcl-xL) and proapoptotic (Bax, Bak, and Bim) proteins [44–49]. Interaction with proapoptotic proteins promotes the release of Cyto C which consequently leads to cell damage and apoptosis [44, 50]. Interaction with the antiapoptotic proteins results in protection against apoptosis [47]. The above-mentioned discussions strongly support the role of VDAC1 in maintaining normal mitochondria function. In our present study, the direct interaction between TPPP3 and VDAC1 was confirmed by Co-IP for the first time. CHX-chase assay and Western blot assay demonstrate that TPPP3 could enhance the stability of VDAC1 (Figures 3(c) and 3(e)). Silencing VDAC1 abolished the effects of TPPP3 on HUVECs under cell stress. The following functional experiments further confirmed that VDAC1 was required for TPPP3-regulated HUVEC injuries.

Hyperlipidemia is known to impair endothelial function in experimental animals and humans [51, 52]. Oxidative stress induced by FFAs plays a critical role in the development of cardiovascular diseases in metabolic syndrome [53] and has been proposed to be a potential pathogenic mechanism linking obesity and insulin resistance [54]. FFAs are used to efficiently generate energy primarily through *β*-oxidation. However, excessive FFAs, along with enhanced oxidation, produce increased ROS and cellular oxidative damage [55]. PA is the primary FFAs in plasma and a common modeling agent for endothelial dysfunction [56]. Excessive generation of ROS can cause cellular injury and dysfunction by directly oxidizing and damaging DNA, proteins, and lipids, as well as by activating several cellular stress-signaling and inflammatory pathways [53]. ROS are mainly produced in the mitochondria. As the most abundant protein of the outer membrane of mitochondria, VDAC1 has been proposed to mediate ROS release from the intermembranal space to the cytosol, thus affecting cell survival [57]. Coincidentally, TPPP3 could interact with VDAC1 and regulate its stability. VDAC1 serves as a bridge to bring TPPP3 and oxidative stress together. In the present study, we demonstrated that PA could induce apoptosis by generating excessive ROS in endothelial cells, which are consistent with previous studies [20, 58, 59]. To further investigate the downstream pathways of ROS, the possible pathways involved in apoptosis such as Bax and BCL-2 were examined. Interestingly, the apoptosis induced by TPPP3 was blocked by ROS scavenger NAC, indicating that TPPP3 might trigger apoptosis by ROS accumulation. Therefore, it was speculated that the impairment of endothelial injury might derive from oxidative stress and subsequent apoptosis signaling pathways.

**Figure 6:**
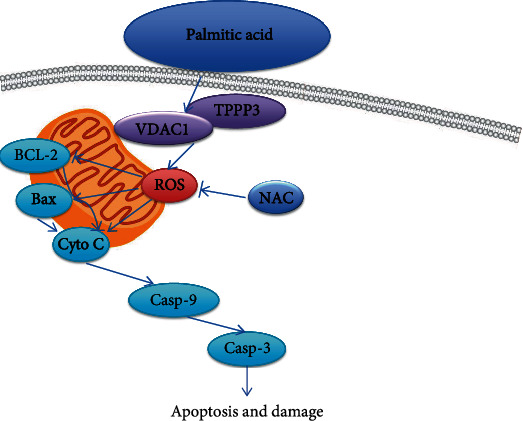
Schematic description of TPPP3, VDAC1, and apoptosis signal pathway.

Understanding how ROS production and scavenging are regulated and developing strategies to decrease intracellular ROS production may have therapeutic potential in the treatment of cardiovascular disease. Our present study showed that TPPP3 played an important role in PA-induced oxidative injury in HUVECs. VDAC1 was identified as a novel interaction partner of TPPP3, and it was demonstrated that TPPP3 might increase VDAC1 stability to trigger ROS production, subsequently activating the apoptosis signaling pathway. Knockdown of VDAC1 or oxidative stress scavenger NAC could abrogate this effect, which further suggested that TPPP3 interacted with VDAC1 to promote PA-induced cell damage via ROS production in HUVECs (Figure 6). However, our data is based on in vitro experiments. In vivo experiments, such as gene-targeting in mice, are needed to clarify the role of TPPP3 as a whole.

## 5. Conclusion

In conclusion, the interaction between TPPP3 and VDAC1 was involved in PA-induced endothelial oxidative injury. TPPP3 promotes PA-induced endothelial oxidative injury by directly interacting with VDAC1. Downregulation of TPPP3 could attenuate PA overload-induced endothelial injury via reducing ROS, which might offer a potential therapeutic target in the treatment for vascular diseases.

## Data Availability

The data used to support the findings of this study are available from the corresponding author upon reasonable request.

## Supplementary Materials

Supplementary Materials Table S1: Potential interacting proteins of TPPP3 found in liquid chromatography/mass spectrometry (LC/MS) analysis.
